# Geriatric nutritional risk index as an independent predictor of 1-year mortality in elderly patients with hip fractures

**DOI:** 10.1371/journal.pone.0348117

**Published:** 2026-06-10

**Authors:** Cengizhan Kurt, Sercan Capkin, Ali Ihsan Kilic, Hakan Cici, Mehmet Akdemir

**Affiliations:** 1 Izmir Bakircay University, Faculty of Medicine, Department of Orthopaedics and Traumatology, Izmir, Turkey; 2 Izmir Democracy University, Faculty of Medicine, Department of Orthopaedics and Traumatology, Izmir, Turkey; 3 Izmir Ekol Hospital, Department of Orthopaedics and Traumatology, Izmir, Turkey; University Hospital of Padova, ITALY

## Abstract

**Background:**

Hip fractures are a major cause of morbidity and mortality in the elderly, with 1-year mortality rates up to 40%. Malnutrition is a key determinant of adverse outcomes. The Geriatric Nutritional Risk Index (GNRI), based on serum albumin and body weight, is a simple nutritional tool. This study investigated whether GNRI independently predicts 1-year mortality after hip fracture surgery beyond conventional risk factors.

**Methods:**

We retrospectively analyzed 624 patients aged ≥65 years who underwent hip fracture surgery at two tertiary centers. Demographic, clinical, and perioperative data were collected. GNRI was calculated for all patients. Independent predictors of mortality were identified using logistic regression, and ROC analysis determined the optimal GNRI cut-off.

**Results:**

The 1-year mortality rate was 22.3%. In univariate analysis, older age, ASA class III–IV, low albumin, low GNRI, and multiple comorbidities were significantly associated with mortality. Each 0.1 g/dL increase in albumin was associated with 26% lower odds of 1-year mortality, and each 1-point increase in GNRI was associated with 25.5% lower odds of mortality (p < 0.001). In multivariate analysis, only age, ASA classification, and GNRI remained independent predictors. ROC analysis showed excellent discrimination for GNRI (AUC: 0.928), with a cut-off of 91.45 (sensitivity 87.8%, specificity 90.3%). Patients with GNRI ≤91.45 had a markedly higher 1-year mortality (72.2% vs. 3.7%; RR: 19.32, 95% CI: 12.01–31.09).

**Conclusions:**

GNRI independently predicted 1-year mortality in elderly hip fracture patients. A GNRI ≤91.45 was associated with a 19-fold higher risk, while higher scores had a protective effect, underscoring GNRI as a practical tool for perioperative risk stratification.

## 1 Introduction

The global elderly population is expanding in parallel with increasing life expectancy, resulting in a concomitant rise in the incidence of hip fractures [1]. Currently, approximately one million hip fractures occur annually worldwide, and this number is projected to increase nearly fivefold by 2050 [1–3]. Hip fractures represent a major cause of morbidity and mortality among older adults [4]. Despite continuous advancements in surgical techniques, perioperative management, and medical care, the 1-year postoperative mortality rate in this population remains unacceptably high, ranging from 20% to 40% [5]. Accordingly, timely identification of risk factors for adverse outcomes is crucial for improving survival and preserving quality of life.

Malnutrition has emerged as a particularly important determinant of poor outcomes in elderly patients with hip fractures [3,6]. It is associated with prolonged hospital stays, higher complication rates, and increased mortality [7]. However, there is no consensus on the most accurate and practical method to assess nutritional status in this population, and existing evaluation tools differ in their predictive validity.

The Geriatric Nutritional Risk Index (GNRI), first proposed by Bouillanne et al. [8], is an objective and simple prognostic tool designed to assess the risk of morbidity and mortality in elderly hospitalized patients. GNRI is calculated using serum albumin levels—a well-established marker of nutritional and inflammatory status—and the ratio of present body weight to ideal body weight, thereby integrating both biochemical and anthropometric parameters [8]. Several recent studies have confirmed GNRI as an independent and robust predictor of both short- and long-term mortality in older patients undergoing hip fracture surgery, underscoring its utility for perioperative risk stratification [9–11].

Nevertheless, evidence is still limited regarding whether GNRI maintains its prognostic value when evaluated alongside other well-established risk factors such as age, sex, fracture type, comorbidity burden, and ASA classification. Therefore, the present study aimed to investigate whether GNRI serves as an independent predictor of 1-year mortality in elderly patients undergoing hip fracture surgery. We hypothesized that lower GNRI scores would remain independently associated with an increased risk of 1-year postoperative mortality, even after adjustment for established demographic and clinical risk factors.

## 2 Materials and methods

### 2.1 Study design and population

This retrospective cohort study included patients aged ≥65 years who underwent surgical treatment for acute traumatic hip fractures between 01/01/2020 and 31/12/2024 at two tertiary care centers, namely Izmir Bakircay University Training and Research Hospital and İzmir Democracy University Training and Research Hospital.

Eligible patients were required to have radiologically confirmed acute hip fractures, surgical treatment with either hemiarthroplasty or proximal femoral nailing, complete clinical records, and known 1-year vital status. Exclusion criteria were age < 65 years (n = 44), pathological fractures (n = 3), periprosthetic fractures (n = 9), multiple trauma (n = 12), previous ipsilateral hip surgery (n = 15), incomplete data or inability to calculate the GNRI (n = 68), and unknown 1-year vital status (n = 11).Of the 786 patients initially identified, 624 were included in the final analysis. Because of the retrospective cohort design, no probability-based sampling strategy was applied and no a priori sample size calculation was performed. Instead, all consecutive eligible patients treated at the participating centers during the predefined study period were included in the analysis.

Ethical approval was obtained from the İzmir Democracy University Ethics Committee (Approval No:2025/505; 2025), and the study was conducted in accordance with the principles of the Declaration of Helsinki.

### 2.2 Data collection

Demographic and clinical variables—including age, sex, fracture type (femoral neck or intertrochanteric), type of anesthesia (regional [spinal or epidural] or general), surgical procedure (hemiarthroplasty or proximal femoral nailing), fracture-to-surgery interval (days), surgery-to-discharge interval (days), and total hospital stay (days)—were extracted from electronic medical records.

Age was categorized into three groups: 65–75, 76–85, and >85 years. Pre-existing comorbidities, including diabetes mellitus; hypertension; coronary artery disease; cerebrovascular disease; chronic obstructive pulmonary disease; chronic renal failure; thyroid disease; anemia; dementia; and malignancy, were recorded individually. The total number of comorbidities was further classified as none, 1–2, or ≥3. Age was categorized to facilitate clinically meaningful stratification of younger-old, middle-old, and oldest-old patients, while also being retained as a continuous variable in regression analyses to avoid loss of information. Similarly, the number of comorbidities was grouped to provide a simple summary of multimorbidity burden for descriptive and univariate analyses.

The American Society of Anesthesiologists (ASA) physical status classification was recorded both as individual grades (I–IV) and as two categories: low-risk (ASA I–II) and high-risk (ASA III–IV).

### 2.3 GNRI assessment

Nutritional status was assessed using the GNRI, calculated as follows [8]: GNRI = (14.89 × serum albumin [g/dL]) + (41.7 × present body weight [kg]/ideal body weight [kg]). Serum albumin, a widely used biochemical marker reflecting both nutritional and inflammatory status, was measured within 24 hours of hospital admission. Ideal body weight was estimated using the Lorentz formula. GNRI values were categorized according to the original classification proposed by Bouillanne et al. [8], which has also been adopted in subsequent studies evaluating nutritional risk in elderly populations: ≥ 98, normal nutritional status; 92–97.9, mild malnutrition risk; 82–91.9, moderate malnutrition risk; and <82, severe malnutrition risk.

### 2.4 Outcome measures

The primary outcome was 1-year all-cause mortality following hip fracture surgery. Mortality data were obtained from hospital records and verified, when necessary, through telephone interviews with patients’ relatives. Mortality was recorded as a binary variable (alive or deceased within 1 year). For deceased patients, the time of death was further categorized into four intervals: 0–3 months, 3–6 months, 6–9 months, and 9–12 months postoperatively. Because the primary endpoint of the study was predefined as 1-year all-cause mortality, the main analyses were based on a binary outcome framework. The timing of death was collected in broad postoperative intervals for descriptive purposes among deceased patients; however, exact time-to-event data were not uniformly available for all participants in a format suitable for formal survival analysis.

### 2.5 Statistical analysis

All statistical analyses were performed using IBM SPSS Statistics for Windows, Version 25.0 (IBM Corp., Armonk, NY, USA). Continuous variables were expressed as mean ± standard deviation (SD), and categorical variables as frequencies and percentages. The normality of continuous variables was assessed using the Shapiro–Wilk test.

Between-group comparisons were conducted using the Student’s t-test for normally distributed continuous variables and Pearson’s chi-square test for categorical variables; Fisher’s exact test was applied in cases where the expected cell count was < 5.

Univariate logistic regression was first performed to screen variables associated with 1-year mortality. For the multivariable model, only preoperative clinically relevant and non-collinear covariates were considered. Assumptions for logistic regression were checked before model construction, including collinearity among candidate predictors and analysis of continuous variables in their original form. Odds ratios (ORs) and 95% confidence intervals (CIs) were calculated for both univariate and multivariate models.

The optimal cut-off value of the GNRI for predicting 1-year mortality was determined using receiver operating characteristic (ROC) curve analysis. The area under the curve (AUC) was calculated, and the Youden index was applied to identify the threshold value. Based on this cut-off, patients were stratified into low and high GNRI groups, and 1-year mortality rates were compared between groups; relative risk and 95% confidence intervals were calculated. Formal survival analysis was not performed because exact event dates and censoring times were not uniformly available for the full cohort; therefore, mortality timing was summarized descriptively.

A p-value < 0.05 was considered statistically significant.

## 3 Results

### 3.1 Baseline characteristics of the study population

**Fig 1:**
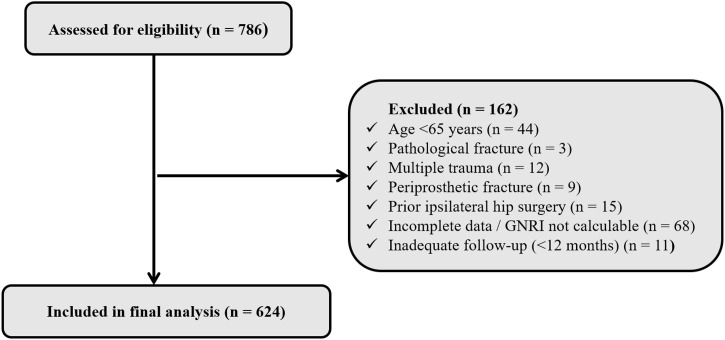
Flow diagram of patient selection and exclusion process for the study.

A total of 786 patients with hip fractures were screened for eligibility. After applying the exclusion criteria, 624 patients were included in the final analysis (Fig 1).

The baseline demographic and clinical characteristics of the study population are presented in Table 1. The mean age was 78.8 ± 7.1 years, and 65.4% of patients were female. When expressed as within-group mortality rates, mortality was highest among patients aged >85 years (63/121, 52.1%), those with ASA III–IV (112/141, 79.4%), those with ≥3 comorbidities (104/173, 60.1%), and those with severe or moderate malnutrition risk (GNRI ≤ 91.9; 122/183, 66.7%). In contrast, mortality was 5.6% in patients with ASA I–II (27/483) and 0.8% in those with normal nutritional status (2/240). Overall, the 1-year mortality rate was 22.3%, and 43.9% of deaths occurred within the first 3 postoperative months. Also, there was no significant association between surgical procedure type and mortality (p = 0.764). The mean serum albumin level was 3.47 ± 0.43 g/dL, and the mean GNRI score was 93.5 ± 6.35. Both lower albumin levels and lower GNRI categories were significantly associated with higher 1-year mortality (p < 0.001); specifically, 66.7% of patients at severe or moderate malnutrition risk (GNRI ≤ 91.9) died within 1 year.

**Table 1:** Baseline demographic, clinical, and nutritional characteristics of the study population (n = 624).

Variable	n/Mean ± SD (range)	Overall %
Age (years), mean ± SD (range)	78.8 ± 7.14 (65–102)	—
Time from fracture to surgery (days), mean ± SD (range)	2.23 ± 1.13 (1–6)	—
Albumin (g/dL), mean ± SD (range)	3.47 ± 0.43 (2.3–4.4)	—
GNRI, mean ± SD (range)	93.5 ± 6.35 (76.0–107.2)	—
Age classification		
65–75 years	226	36.2
76–85 years	277	44.4
> 85 years	121	19.4
Sex		
Female	408	65.4
Male	216	34.6
Type of hip fracture		
Femoral neck	349	55.9
Intertrochanteric	275	44.1
ASA classification		
ASA I–II (low-risk)	483	77.4
ASA III–IV (high-risk)	141	22.6
Surgical procedure		
Hemiarthroplasty	495	79.3
Proximal Femoral Nail (PFN)	129	20.7
Number of comorbidities		
None	124	19.9
1–2 comorbidities	327	52.4
≥ 3 comorbidities	173	27.7
GNRI category		
< 82, Severe malnutrition risk	79	12.7
82–91.9, Moderate malnutrition risk	104	16.7
92–97.9, Mild malnutrition risk	201	32.2
≥ 98, Normal nutritional status	240	38.5

The distribution of pre-existing comorbidities in the study population is summarized in Table 2. The most common conditions were hypertension (46.2%) and diabetes mellitus (28.7%), followed by dementia (15.9%) and anemia (11.4%). Several comorbidities—including hypertension, coronary artery disease, cerebrovascular disease, chronic obstructive pulmonary disease, chronic renal failure, anemia, dementia, and malignancy—were significantly associated with increased 1-year mortality (all p < 0.05). In contrast, diabetes mellitus (p = 0.811) and thyroid disease (p = 0.623) showed no significant relationship with mortality.

**Table 2:** Comparison of baseline characteristics and pre-existing comorbidities between survivors and non-survivors within 1 year after hip fracture surgery.

Variable	Overall (n = 624)	Survival group (n = 485)	Death group (n = 139)	p-value
**Age (years), mean ± SD**	78.8 ± 7.14	77.3 ± 6.57	83.9 ± 6.69	<0.001
**Time from fracture to surgery (days), mean ± SD**	2.23 ± 1.13	2.13 ± 1.02	2.55 ± 1.40	<0.001
**Time from surgery to discharge (days), mean ± SD**	6.06 ± 2.70	5.76 ± 2.49	7.11 ± 3.13	<0.001
**Total hospital stay (days), mean ± SD**	8.30 ± 3.13	7.91 ± 2.89	9.67 ± 3.55	<0.001
**Albumin (g/dL), mean ± SD**	3.47 ± 0.43	3.64 ± 0.30	2.91 ± 0.33	<0.001
**GNRI, mean ± SD**	93.5 ± 6.35	95.89 ± 4.5	85.05 ± 4.88	<0.001
**Age classification, n (%)**				<0.001
65–75 years	226 (36.2%)	206 (42.5%)	20 (14.4%)	
76–85 years	277 (44.4%)	221 (45.6%)	56 (40.3%)	
> 85 years	121 (19.4%)	58 (12.0%)	63 (45.3%)	
**Sex, n (%)**				0.234
Female	408 (65.4%)	323 (66.6%)	85 (61.2%)	
Male	216 (34.6%)	162 (33.4%)	54 (38.8%)	
**Type of hip fracture, n (%)**				0.023
Femoral neck	349 (55.9%)	283 (58.4%)	66 (46.1%)	
Intertrochanteric	275 (44.1%)	202 (41.6%)	73 (53.9%)	
**ASA physical status, n (%)**				<0.001
ASA I	88 (14.1%)	83 (18.1%)	5 (3.0%)	
ASA II	394 (63.1%)	373 (76.9%)	21 (14.5%)	
ASA III	109 (17.5%)	21 (4.3%)	88 (62.4%)	
ASA IV	33 (5.3%)	8 (1.7%)	25 (20.0%)	
**ASA classification, n (%)**				<0.001
ASA I–II (low-risk)	483 (77.4%)	456 (94.0%)	27 (18.2%)	
ASA III–IV (high-risk)	141 (22.6%)	29 (6.0%)	112 (81.8%)	
**Type of anesthesia, n (%)**				0.068
Spinal/Epidural	536 (85.9%)	410 (84.5%)	126 (90.6%)	
General anesthesia	88 (14.1%)	75 (15.5%)	13 (9.4%)	
**Surgical procedure, n (%)**				0.764
Hemiarthroplasty	495 (79.3%)	386 (79.6%)	109 (78.4%)	
Proximal Femoral Nail (PFN)	129 (20.7%)	99 (20.4%)	30 (21.6%)	
**Number of comorbidities, n (%)**				<0.001
No comorbidity	124 (19.9%)	119 (24.5%)	5 (3.6%)	
1–2 comorbidities	327 (52.4%)	297 (61.2%)	30 (21.6%)	
≥ 3 comorbidities	173 (27.7%)	69 (14.2%)	104 (74.8%)	
**GNRI category, n (%)**				<0.001
< 82, Severe malnutrition risk	79 (12.7%)	26 (5.4%)	53 (38.1%)	
82–91.9, Moderate malnutrition risk	104 (16.7%)	35 (7.2%)	69 (49.6%)	
92–97.9, Mild malnutrition risk	201 (32.2%)	186 (38.4%)	15 (10.8%)	
≥ 98, Normal nutritional status	240 (38.5%)	238 (49.0%)	2 (1.4%)	
**Pre-existing comorbidities, n (%)**				
DM	179 (28.7%)	138 (28.5%)	41 (29.5%)	0.811*
HT	288 (46.2%)	180 (37.1%)	108 (77.7%)	<0.001*
CAD	88 (14.1%)	38 (7.8%)	50 (36.0%)	<0.001*
CVD	57 (9.1%)	26 (5.4%)	31 (22.3%)	<0.001*
COPD	60 (9.6%)	37 (7.6%)	23 (16.5%)	0.002*
CRF	58 (9.3%)	29 (6.0%)	29 (20.9%)	<0.001*
Thyroid disease	32 (5.1%)	26 (5.4%)	6 (4.3%)	0.623**
Anemia	71 (11.4%)	31 (6.4%)	40 (28.8%)	<0.001*
Dementia	99 (15.9%)	47 (9.7%)	52 (37.4%)	<0.001*
Malignancy	33 (5.3%)	20 (4.1%)	13 (9.4%)	0.015**

Note. Continuous variables were compared using independent samples t-test; categorical variables were compared using Chi-square test.

* Pearson’s chi-square test.

** Fisher’s exact test.

GNRI: Geriatric Nutritional Risk Index; ASA: American Society of Anesthesiologists physical status classification; PFN: Proximal Femoral Nail; DM: diabetes mellitus; HT: hypertension; CAD: coronary artery disease; CVD: cerebrovascular disease; COPD: chronic obstructive pulmonary disease; CRF: chronic renal failure.

A comparison of baseline characteristics between survivors and non-survivors is presented in Table 2. Patients in the mortality group were significantly older (83.9 ± 6.69 vs. 77.3 ± 6.57 years; p < 0.001), had longer fracture-to-surgery intervals (2.55 ± 1.40 vs. 2.13 ± 1.02 days; p < 0.001), longer surgery-to-discharge durations (7.11 ± 3.13 vs. 5.76 ± 2.49 days; p < 0.001), and longer total hospital stays (9.67 ± 3.55 vs. 7.91 ± 2.89 days; p < 0.001). The mean serum albumin level was markedly lower in the mortality group (2.91 ± 0.33 vs. 3.64 ± 0.30 g/dL; p < 0.001), and the mean GNRI score was also significantly lower (85.05 ± 4.88 vs. 95.89 ± 4.50; p < 0.001). Non-survivors were more likely to be > 85 years of age (45.3% vs. 12.0%; p < 0.001), to have intertrochanteric fractures (53.9% vs. 41.6%; p = 0.023), and to present with ASA class III–IV (81.8% vs. 6.0%; p < 0.001). The proportion of patients with ≥3 comorbidities (74.8% vs. 14.2%; p < 0.001) and those at severe or moderate malnutrition risk (87.7% vs. 12.6%; p < 0.001) was also markedly higher among non-survivors. No significant differences were observed with respect to sex, anesthesia type, or surgical procedure.

### 3.2 Univariate logistic regression analysis

In univariable logistic regression analyses, older age, longer fracture-to-surgery interval, longer postoperative hospital stay, lower albumin, lower GNRI, intertrochanteric fracture type, higher ASA status, and greater comorbidity burden were associated with higher 1-year mortality (Table 3). Sex, anesthesia type, and surgical procedure were not significantly associated with mortality. Very high odds ratios observed in the lowest GNRI categories should be interpreted with caution, as the reference group (GNRI ≥98) included only two deaths (Table 3).

**Table 3:** Univariable and multivariable logistic regression analyses for predictors of 1-year mortality after hip fracture.

Variable	Univariable OR (95% CI)	p-value	Multivariable adjusted OR (95% CI)	p-value
Age (per 1-year increase)	1.156 (1.119–1.195)	<0.001	1.079 (1.030–1.130)	0.001
Time from fracture to surgery (per 1-day increase)	1.368 (1.165–1.607)	<0.001	—	—
Time from surgery to discharge (per 1-day increase)	1.188 (1.110–1.271)	<0.001	—	—
Total hospital stay (per 1-day increase)	1.190 (1.120–1.265)	<0.001	—	—
GNRI (per 1-point increase)	0.745 (0.714–0.778)	<0.001	0.849 (0.803–0.897)	<0.001
Albumin (per 0.1 g/dL increase)	0.74 (0.69–0.78)	<0.001	—	—
Age classification		<0.001	—	—
76–85 years vs 65–75 years	2.610 (1.514–4.500)	0.001	—	—
> 85 years vs 65–75 years	11.188 (6.256–20.008)	<0.001	—	—
Sex			—	—
Female vs Male	0.789 (0.535–1.166)	0.234	—	—
Type of hip fracture			—	—
Intertrochanteric vs Femoral neck	1.550 (1.061–2.263)	0.023	—	—
ASA physical status		<0.001	—	—
ASA II vs ASA I	0.935 (0.342–2.550)	0.895	—	—
ASA III vs ASA I	69.562 (25.074–192.980)	<0.001	—	—
ASA IV vs ASA I	51.875 (15.570–172.835)	<0.001	—	—
ASA classification				
ASA III–IV vs ASA I–II	65.226 (37.130–114.583)	<0.001	10.770 (5.205–22.288)	<0.001
Type of anesthesia			—	—
General vs Spinal/Epidural	0.564 (0.303–1.050)	0.071	—	—
Surgical procedure			—	—
PFN vs Hemiarthroplasty	1.073 (0.677–1.701)	0.764	—	—
Number of comorbidities		<0.001	—	—
1–2 vs none	2.404 (0.911–6.344)	0.076	—	—
≥ 3 vs none	35.872 (13.942–92.301)	<0.001	—	—
GNRI category		<0.001	—	—
< 82 vs ≥ 98	242.577 (55.847–1053.660)	<0.001	—	—
82–91.9 vs ≥ 98	234.600 (55.034–1000.057)	<0.001	—	—
92–97.9 vs ≥ 98	9.597 (2.168–42.489)	0.003	—	—

**Note**. OR: Odds ratio; CI: Confidence interval; GNRI: Geriatric Nutritional Risk Index; ASA: American Society of Anesthesiologists physical status classification; PFN: Proximal Femoral Nail. Continuous variables were analyzed per unit increase. Only preoperative, clinically relevant, and non-collinear covariates were included in the multivariable model. Postoperative variables, such as time from surgery to discharge and total hospital stay, were excluded because they reflect the subsequent clinical course rather than baseline risk. Albumin was not entered together with GNRI because of conceptual and statistical overlap. ASA classification was compared with I–II as the reference group.

### 3.3 Multivariate logistic regression analysis

In the multivariable logistic regression model, which was restricted to preoperative, clinically relevant, and non-collinear covariates, age, ASA classification, and GNRI remained independently associated with 1-year mortality. Anesthesia type was not significantly associated with 1-year mortality in either the unadjusted group comparison (p = 0.068) or univariate logistic regression analysis (p = 0.071). Each additional year of age was associated with a 7.9% increase in the odds of mortality (OR: 1.079; 95% CI: 1.030–1.130; p = 0.001). Patients classified as ASA III–IV had 10.77-fold higher odds of 1-year mortality compared with those classified as ASA I–II (OR: 10.770; 95% CI: 5.205–22.288; p < 0.001). Higher GNRI values were independently protective, with each 1-point increase associated with 15.1% lower odds of mortality (OR: 0.849; 95% CI: 0.803–0.897; p < 0.001).

### 3.4 ROC curve analysis and GNRI cut-off value

**Fig 2:**
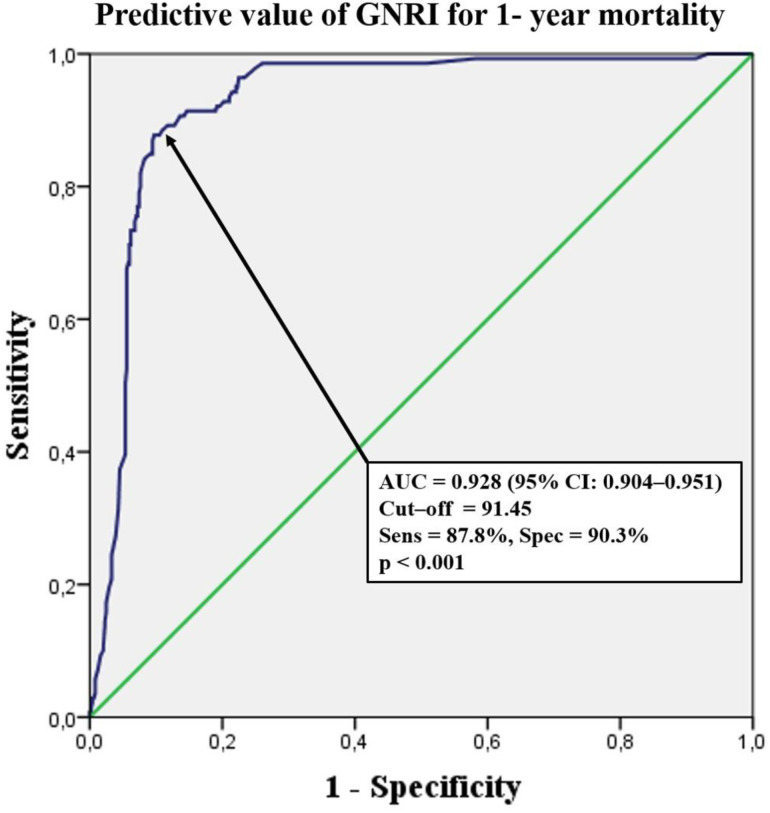
ROC curve showing the predictive performance of GNRI for 1-year mortality after hip fracture surgery.

ROC analysis showed that the GNRI had excellent discriminatory power for predicting 1-year mortality after hip fracture, with an AUC of 0.928 (95% CI: 0.904–0.951; p < 0.001). The optimal cut-off value, determined using the Youden Index, was 91.45, providing a sensitivity of 87.8% and a specificity of 90.3% (Youden Index = 0.781) (Fig 2).

Patients with a GNRI ≤91.45 experienced nearly a 20-fold higher risk of 1-year mortality compared with those with a GNRI >91.45 (72.2% vs. 3.7%; RR: 19.32, 95% CI: 12.01–31.09; p < 0.001).

## 4 Discussion

In this retrospective, two-center cohort study of elderly patients undergoing surgery for acute hip fractures, three independent predictors of 1-year mortality were identified: age, ASA classification, and GNRI. Multivariate logistic regression demonstrated that each additional year of age increased mortality odds by 7%, while ASA class III–IV patients had more than a ten-fold higher odds compared with ASA class I–II. Moreover, each 1-point increase in GNRI was associated with a 15% reduction in mortality odds. Consistently, using a clinically relevant threshold, patients with GNRI ≤ 91.45 had nearly a 19-fold higher 1-year mortality risk compared with those above this cut-off. Although serum albumin also emerged as a strong predictor in the univariate analysis, it was excluded from the multivariate model due to collinearity with GNRI, which inherently incorporates albumin as a core component and thus provides a more comprehensive prognostic measure. These findings emphasize that, beyond chronological age and physiological reserve, preoperative nutritional status—as captured by GNRI—provides important prognostic information, supporting its potential as a practical and objective tool for perioperative risk stratification.

The GNRI, originally proposed by Bouillanne et al. [8], is an objective index incorporating serum albumin and body weight ratio to predict morbidity and mortality in elderly populations. In their cohort, lower GNRI values were strongly associated with adverse outcomes, with GNRI ≤ 82 linked to up to 29-fold increased mortality odds [8]. In line with this, our analysis showed that patients with GNRI < 82 had a markedly elevated 1-year mortality odds (up to 243-fold) compared with those with GNRI ≥98. This striking difference may be explained by the older mean age of our cohort, the higher prevalence of ASA class III–IV, greater multimorbidity, frequent cardiovascular and cerebrovascular comorbidities, and a considerable early postoperative mortality rate (43.9% of deaths occurred within 3 months). It should be noted, however, that the extremely high odds ratios observed for the lowest GNRI categories (e.g., < 82) likely reflect the very small number of deaths in the reference group (GNRI ≥98, n = 2), which may have inflated relative odds estimates and widened confidence intervals. Therefore, while the direction of the association is robust and consistent with prior studies, the absolute magnitude of these odds ratios should be interpreted with caution. Collectively, these findings reinforce GNRI as a valuable prognostic marker in elderly hip fracture patients.

Previous studies have also reported the prognostic utility of GNRI in predicting short-term mortality after hip fracture surgery [12,13]. For example, in a retrospective cohort of 1040 patients, GNRI was identified as a significant predictor of 30-day mortality, with ROC analysis yielding an optimal cut-off of 75.4; patients below this threshold had up to 27-fold increased mortality odds. This underscores the usefulness of GNRI in the immediate postoperative period. Extending beyond these findings, our study demonstrated that GNRI independently predicts not only early but also longer-term mortality, with an optimal cut-off of 91.45 below which patients had a nearly 19-fold increased risk. Moreover, a comparative study assessing several nutritional assessment tools—including Graz Malnutrition Screening, Prognostic Nutritional Index, GNRI, and Controlling Nutritional Status—found only limited predictive value across all scores (AUC = 0.64–0.68), with no clear superiority [13]. By contrast, our ROC analysis showed that GNRI alone provided excellent discriminatory ability for predicting 1-year mortality (AUC = 0.928), underscoring its robustness as a prognostic marker.

Yerli et al. further evaluated GNRI in relation to 90-day outcomes in a large cohort of 1345 elderly hip fracture patients [14]. They reported a 90-day mortality rate of 10.6%, with GNRI significantly associated with short-term mortality (p < 0.001) but not with early postoperative complications. However, ROC analysis to define an optimal cut-off was not performed. While their findings highlight the role of GNRI in predicting early mortality, our study expands on this by demonstrating its strong association for 1-year mortality. Specifically, we observed a higher mortality rate of 22.3% and identified an optimal GNRI threshold of 91.45, with excellent discriminatory performance (AUC = 0.928).

Similarly, Kotera et al. analyzed 607 patients (mean age 87 ± 6 years) and reported a 180-day mortality rate of 5.4% [11]. Non-survivors had significantly lower mean GNRI values (83 ± 9) than survivors (92 ± 9), with ROC analysis showing moderate discriminatory power (AUC = 0.74). In our study, the mean GNRI was 85.05 ± 4.88 in the death group and 95.89 ± 4.50 in the survivor group, with a substantially higher 1-year mortality rate of 22.3%. Importantly, our higher optimal cut-off (91.45) demonstrated excellent predictive accuracy (AUC = 0.928). These findings indicate that GNRI is not only a predictor of short- to mid-term outcomes, as suggested by Kotera et al., but also a robust marker of long-term mortality. Moreover, Yokoyama et al. [15] reported a 6-month mortality rate of 13.3% in patients with GNRI < 92 undergoing bipolar hemiarthroplasty, also noting higher complication, transfusion, and impaired gait rates. Compared with their findings, our study demonstrated a higher mortality rate, likely reflecting the longer follow-up, larger sample, and more comorbid cohort.

Furthermore, Fujimoto et al. [10], Wu et al. [9], and Liu et al. [16] consistently emphasized the prognostic significance of GNRI in hip fracture patients. Fujimoto et al. reported that each 1-point increase in GNRI reduced 1-year mortality odds by 20% [10]; Wu et al. found that low GNRI nearly doubled 1-year mortality odds and was associated with higher early postoperative complications [9]; and Liu et al., in a meta-analysis of 3959 patients, confirmed significant associations between lower GNRI, increased mortality, and higher complication rates [16]. In our two-center cohort of 624 patients, a consistent inverse association was observed, with each 1-point increase in GNRI reducing 1-year mortality odds by 15% (OR: 0.85; 95% CI: 0.81–0.91; p < 0.001). The optimal GNRI cut-off of 91.45 also effectively stratified mortality risk. Collectively, evidence from single-center, multi-institutional, and pooled analyses converges to support GNRI as a strong and independent predictor of 1-year mortality after hip fracture, reinforcing its potential role in preoperative risk stratification and in guiding targeted nutritional and multidisciplinary interventions.

In contrast, Choi et al. [17] conducted a study involving 548 patients aged ≥80 years who underwent surgery for proximal femur fractures, focusing exclusively on an “extremely elderly” population and evaluating major morbidities (including in-hospital mortality) as the primary endpoint. They reported that GNRI was not a significant predictor of morbidity (AUC = 0.561, p = 0.083), whereas serum albumin level (cut-off: 3.55 g/dL) and the Charlson Comorbidity Index (CCI) emerged as independent predictors. The overall major morbidity rate was 14.4%, and the in-hospital mortality rate was 4% [17]. By contrast, our study encompassed a broader age range (65–102 years), included patients from two centers, and focused on long-term outcomes with 1-year mortality as the primary endpoint. We observed a 1-year mortality rate of 22.3%, with 43.9% of deaths occurring within the first 3 months after surgery. GNRI was confirmed as an independent prognostic factor, and patients with GNRI ≤91.45 exhibited a 19-fold higher risk of death. Moreover, ROC analysis demonstrated excellent discriminative ability of GNRI for predicting 1-year mortality (AUC = 0.928). Taken together, while the findings of Choi et al. suggest limitations of GNRI in predicting short-term morbidity in extremely elderly patients, our results highlight its strong prognostic value for long-term mortality across a broader elderly population. This discrepancy is likely attributable to differences in follow-up duration (in-hospital vs. 1-year), the predominance of early mortality, variations in patient age distribution, and the choice of outcome measures (morbidity vs. mortality).

Additionally, Zhou et al. [18] proposed a novel combined index integrating GNRI with the Systemic Immune-inflammatory Index (SII) in a cohort of 597 elderly hip fracture patients, reporting an overall 1-year mortality of 15.1%. Patients with high SII–GNRI scores exhibited a substantially higher mortality rate (28.8%) compared with those with low scores (7.6%). This study highlights the prognostic significance of simultaneously considering malnutrition and systemic inflammation, suggesting that the integration of GNRI and SII may offer a more comprehensive and accurate tool for risk stratification. Taken together, the available evidence indicates that while GNRI alone remains a robust predictor of long-term mortality, its predictive value may be further enhanced when combined with markers of systemic inflammation.

Importantly, while our findings highlight the prognostic value of GNRI in predicting post-fracture mortality, a recent large-scale prospective study by Pan et al. [19] further demonstrated its association with fragility fracture risk in elderly patients with type 2 diabetes mellitus. In this 9-year ambispective cohort, lower GNRI levels were significantly linked to an increased incidence of fragility fractures, as well as to higher 10-year probabilities of major osteoporotic and hip fractures assessed by the Fracture Risk Assessment Tool (FRAX) [19]. Similarly, Wang et al. [20], analyzing data from the U.S. National Health and Nutrition Examination Survey (NHANES), reported that higher GNRI values were positively associated with femoral bone mineral density and negatively associated with the risk of osteoporosis in postmenopausal women. Consistently, Ji et al. [21] found that lower GNRI was independently associated with osteoporosis among elderly patients with type 2 diabetes in Northern China.

Moreover, Yoshida et al. [22] demonstrated that a low GNRI was an independent risk factor for bone fractures in patients undergoing hemodialysis, with fracture incidence being significantly higher in the low GNRI group regardless of sex. These results suggest that GNRI may serve not only as a prognostic marker after fracture but also as a predictor of bone health and fracture occurrence, underscoring its role as a comprehensive biomarker. In line with these findings, Kamioka et al. [23] showed that lower GNRI was significantly associated with osteoporosis and higher FRAX-derived fracture risk in patients with chronic liver disease, further reinforcing its value as a simple and reliable indicator of bone health.

Taken together with our findings, this growing body of evidence supports the potential of GNRI to contribute both to preventive strategies and to post-fracture mortality risk stratification in elderly populations. Nonetheless, further studies in larger and more diverse cohorts are warranted to confirm its generalizability.

This study has several strengths, including its relatively large sample size, two-center design, and comprehensive statistical analyses incorporating both multivariate regression and ROC curve evaluation, which together provide robust evidence for the prognostic utility of GNRI in elderly hip fracture patients. Nevertheless, certain limitations should be acknowledged. The retrospective design may have introduced selection bias, and the study population was regionally restricted, which may limit the generalizability of our findings. Measurement bias is also possible because some variables were obtained from routine medical records. In addition, potentially important prognostic factors, including sarcopenia, pre-fracture mobility, and cognitive status, were not uniformly available and could not be analyzed. Formal survival analysis was not performed because exact event dates and censoring times were not uniformly available for the full cohort; therefore, mortality timing was summarized descriptively. Moreover, other nutritional indices and postoperative complications were not systematically evaluated, precluding direct comparison of GNRI with alternative prognostic tools, and important confounding factors such as frailty, pre-fracture functional status, and inflammatory markers were not included in the analysis. The GNRI cut-off was derived from the same dataset and was not internally and externally validated therefore, the threshold should be interpreted as exploratory and cohort-specific.

Future large-scale, prospective, multi-institutional studies are warranted to validate our results, to assess the additive value of GNRI when combined with functional and inflammatory parameters, and to explore whether GNRI-guided nutritional and multidisciplinary interventions can improve survival outcomes in this vulnerable population.

## 5 Conclusions

This two-center retrospective cohort study demonstrated that GNRI is an independent and robust predictor of 1-year mortality in elderly patients undergoing surgery for acute hip fractures. A GNRI ≤91.45 was associated with nearly a 19-fold higher risk of death, while higher scores were protective. These findings highlight the importance of incorporating GNRI into routine preoperative assessment, as patients with low GNRI may benefit from closer monitoring and targeted multidisciplinary interventions. Future prospective studies are warranted to validate these results and to determine whether GNRI-guided management can improve survival outcomes in this vulnerable population.

## Abbreviations

ASA: American Society of Anesthesiologists (score/classification)
AUC: Area Under the Curve
CAD: Coronary Artery Disease
CCI: Charlson Comorbidity Index
CI: Confidence Interval
COPD: Chronic Obstructive Pulmonary Disease
CRF: Chronic Renal Failure
CVD: Cerebrovascular Disease
DM: Diabetes Mellitus
FRAX: Fracture Risk Assessment Tool
GNRI: Geriatric Nutritional Risk Index
HT: Hypertension
NHANES: National Health and Nutrition Examination Survey
OR: Odds Ratio
ROC: Receiver Operating Characteristic
SD: Standard Deviation
SII: Systemic Immune-Inflammation Index
SPSS: Statistical Package for the Social Sciences
RR: Relative Risk
